# Early Developing Pig Embryos Mediate Their Own Environment in the Maternal Tract

**DOI:** 10.1371/journal.pone.0033625

**Published:** 2012-03-28

**Authors:** Carmen Almiñana, Paul R. Heath, Stephen Wilkinson, Jonatan Sanchez-Osorio, Cristina Cuello, Inmaculada Parrilla, Maria A. Gil, Jose L. Vazquez, Juan Maria Vazquez, Jordi Roca, Emilio A. Martinez, Alireza Fazeli

**Affiliations:** 1 Academic Unit of Reproductive and Developmental Medicine, Department of Human Metabolism, The University of Sheffield, Sheffield, United Kingdom; 2 Sheffield Institute for Translational Neuroscience (SITraN), Sheffield, United Kingdom; 3 Chemical and Biological Engineering, The University of Sheffield, Sheffield, United Kingdom; 4 Department of Medicine and Animal Surgery, Veterinary Faculty, University of Murcia, Murcia, Spain; Institut Jacques Monod, France

## Abstract

The maternal tract plays a critical role in the success of early embryonic development providing an optimal environment for establishment and maintenance of pregnancy. Preparation of this environment requires an intimate dialogue between the embryo and her mother. However, many intriguing aspects remain unknown in this unique communication system. To advance our understanding of the process by which a blastocyst is accepted by the endometrium and better address the clinical challenges of infertility and pregnancy failure, it is imperative to decipher this complex molecular dialogue. The objective of the present work is to define the local response of the maternal tract towards the embryo during the earliest stages of pregnancy. We used a novel in vivo experimental model that eliminated genetic variability and individual differences, followed by Affymetrix microarray to identify the signals involved in this embryo-maternal dialogue. Using laparoscopic insemination one oviduct of a sow was inseminated with spermatozoa and the contralateral oviduct was injected with diluent. This model allowed us to obtain samples from the oviduct and the tip of the uterine horn containing either embryos or oocytes from the same sow. Microarray analysis showed that most of the transcripts differentially expressed were down-regulated in the uterine horn in response to blastocysts when compared to oocytes. Many of the transcripts altered in response to the embryo in the uterine horn were related to the immune system. We used an *in silico* mathematical model to demonstrate the role of the embryo as a modulator of the immune system. This model revealed that relatively modest changes induced by the presence of the embryo could modulate the maternal immune response. These findings suggested that the presence of the embryo might regulate the immune system in the maternal tract to allow the refractory uterus to tolerate the embryo and support its development.

## Introduction

Despite many advances in assisted reproductive technologies (ART) in humans, to date the incidence of pregnancy failure remains high [1]. Pregnancy loss also affects livestock breeding and has economical implications for the livestock industry [2]. The high rate of pregnancy failure has been attributed mainly to asynchronous embryo development in the maternal tract and inappropriate communication between the mother and the developing embryo(s) [3], [4], [5]. In addition, with the recent emergence of the field of “Developmental Origins of Health and Disease” (DOHaD), it is becoming increasingly evident that a greater understanding of the milieu of the female reproductive tract and the potential effect that disturbances of this milieu may have on the offspring is needed [6].

The maternal tract hosts a crucial cross-talk between the mother and the embryo at a very early stage of life and it seems that this cross talk may have long term consequences which extend into adulthood [7], [8], [9], [10]. Hence, understanding the complex molecular dialogue between the maternal tract and the embryo is essential for solving infertility problems, reducing pregnancy loss and even identifying the factors that may influence the developmental potential of offspring into adulthood, in human and livestock.

The response of the endometrium towards the embryo(s) at very early stages of pregnancy is poorly understood. A number of genes and proteins have already been described to be activated in the endometrium when the embryo arrives in the maternal tract [5], [11]. It is apparent that precise recognition of the embryo in the maternal tract is critical for preparation of a suitable environment for implantation, embryonic development and pregnancy [12]. However, exactly when the oviduct and the uterus recognize the presence of embryo(s) and how the maternal tract alters its environment in response to embryo(s) is not completely understood.

In the past, it has been generally assumed that maternal-embryo communication and the environment of the womb are mainly controlled by the endocrine system [13], [14]. Many studies have compared genomic and proteomic profiles of pregnant and non-pregnant animals, pointing to major differences in transcriptional activities that are mainly under hormonal control (mouse: [10], [15]; bovine: [16], [17], [18]; pig [19], [20], [21]; human: [1], [22]). Paracrine and autocrine signals from the embryo and the maternal tract are also involved in this important process [23], [24], [25]. This complex network of signals is reflected in alteration of uterine transcriptome, leading to morphological, biochemical and also immunological changes in the uterine environment.

Little is known about the potential local signalling molecules involved in cell-to-cell interactions in embryo-maternal communication. In literature, only one study has provided evidence of transcriptional changes in the oviduct in response to the embryo that can be regarded as maternal tract responses to the local signals received from embryo and not related to the hormones released during pregnancy [10]. Technological, experimental and ethical issues make it very difficult to define a clear picture of the local maternal tract responses that are produced due to exactly local signalling by embryo(s) and its arrival in the female reproductive tract.

In the current investigation, we aimed to define the local response(s) of the maternal tract towards the embryo during the very earliest stages of pregnancy. We used a novel porcine *in vivo* experimental model in which each sow was subjected to laparoscopic insemination procedures. One oviduct was inseminated with spermatozoa and the contralateral oviduct was injected with diluent only. This allowed us to obtain samples from the oviduct and the tip of the uterine horn containing either embryos or oocytes. Using this model, the uterine transcriptomic profile in response to the embryo was directly compared to the transcriptomic profile of the uterine horn stimulated by oocytes from the same animal. This model minimized individual variability and allowed comparison of the local responses of the female reproductive tract towards oocyte and embryo under similar hormonal, nutritional, health and environmental conditions.

In addition, to gain more comprehensive understanding of the interactions between the female reproductive tract and the embryo we have used a computational model. Biologically based computational models are becoming crucial for a rigorous analysis of complex signalling networks in different research areas [26], [27]. These models provide us with a detailed insight into specific pathway dynamics. Maternal embryo communication has been described as a complex system in which a broad range of signals are exchanged between the mother and the embryo. To evaluate the full range of feedback signals and resulting effects in pregnancy is unfeasible without the help of analytical computational tools. No *in silico* models of the events in relation to maternal responses to embryo are available up to date. To extract the maximum amount of information from our experimental measures, we have used a published computational model of Toll-like receptor (TLR) 4 [28] based on the data obtained in our experiments together with the current biological knowledge of the TLR pathway. This approach helps us to understand the complex maternal-embryo interactions.

Here, we reported that the pig embryos at the blastocyst stage elicit a local response in the sow endometrium. Many of the genes altered in the uterine horn in response to the embryo were related to the immune system. This implied that the presence of the embryo in the maternal tract regulated local immune responses and potentially reduced the local activity of the innate immune system within the maternal tract at the site of implantation, preparing it for implantation to take place. Furthermore, *in silico* modelling results revealed that relatively modest changes induced by the presence of the embryo could modulate the maternal immune response. These findings suggested that the presence of the embryo might regulate the immune system in the maternal tract to allow the refractory uterus to tolerate the embryo and support its development.

## Materials and Methods

### Animals

Weaned crossbred sows from Landrace x Large White genetic line (from two to six parities) were selected to perform the experiments. Sows were housed individually in an environmentally controlled unit in a mechanically ventilated confinement facility under field conditions in a commercial pig farm. Sows were fed a commercial ration twice a day and water was provided ad libitum. All experiments were performed after obtaining ethical committee approval from the Ethical Committee for Experimentation with Animals of the University of Murcia, Spain (385/2008). All laparoscopic inseminations and surgeries were performed under anaesthesia, and all efforts were made to minimize suffering.

### Detection of oestrus and ovarian status

Oestrus detection was carried out once a day, 2 days after weaning, by exposing females to a mature boar and applying manual back-pressure. Females that showed a standing oestrus reflex were considered to be in heat and the ovaries were scanned. The ovaries were examined by transrectal ultrasonography (Pie Medical SC100 Scanner, Maastricht, The Netherlands) 12 h after the onset of oestrus and just before the insemination (36 h after onset of estrus) using a 7.5 MHz multiple scan angle transducer as described by Soede et al. [29], and modified by Bolarin and colleagues [30]. Only sows showing multiple pre-ovulatory follicles (diameter of antrum >6 mm) were selected for experiments. Inseminations were carried out within 2–3 h after the ultrasonography.

### Semen Collection and evaluation

Three sexually mature hybrid boars of proven fertility were selected for semen collection. The sperm-rich fraction of the ejaculates was collected using the gloved hand manual method. Pooled semen was extended in Beltsville Thawing solution (BTS; [31]) to 30×10^6^ spermatozoa/ml (Inseminate doses). After collection, seminal characteristics (total sperm numbers per ejaculate, subjective sperm motility, acrosome integrity and normal morphology) were evaluated using standard laboratory techniques [32]. Before laparoscopic insemination, insemination doses were diluted in BTS to 3×10^5^ spermatozoa/100 µl.

### Intraoviductal laparoscopic insemination

**Figure 1:**
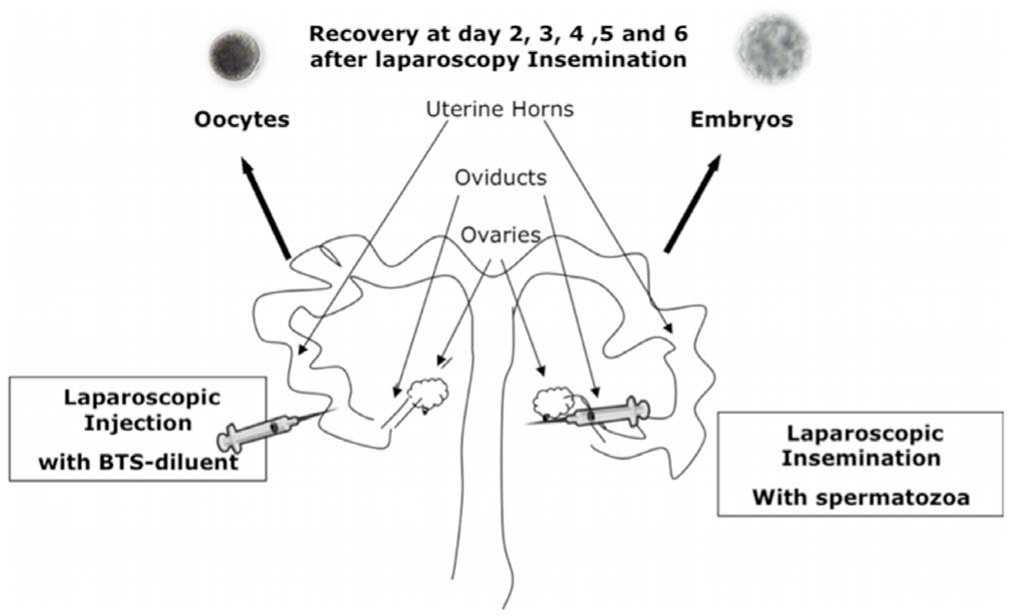
Schematic representation of the experimental design. Sows were subjected to laparoscopic surgery. While one oviduct was subjected to laparoscopic insemination with (3×10^5^ spermatozoa/100 µl) spermatozoa (inseminated side), the contralateral oviduct was injected with BTS-diluent containing no sperm (non inseminated side). Oviductal and uterine horn samples as well as flushings were collected from both horns at day 2, 3, 4, 5 and 6 after laparoscopy insemination by hysterectomy. The presence of embryos at different stages of pregnancy in one horn (inseminated side), and the existence of unfertilized oocytes in the other horn (non inseminated side) were verified by careful examination of flushings under a stereomicroscope.

Laparoscopic inseminations were performed on sows sedated by azaperone administration (2 mg/kg body weight, i.m.). General anesthesia was induced with sodium thiopenthal (7 mg/kg body weight, i.v.) and maintained with isofluorane (3.5–5%). Each sow undergoing anesthetic procedures was placed in the supine position and a pneumoperitoneum was established. The abdominal cavity was insufflated with CO_2_ to 14 mmHg. Two accessory ports were placed in the right and left part of the hemi abdomen, which provided access for laparoscopic Duval forceps for manipulating the uterine horn and grasping the oviduct for the insemination, respectively. The oviduct was grasped with the Duval forceps in the fundibulum area close to the ampullar region. Then the sperm dose was injected above of the ampullar region in direction to isthmus (3×10^5^ spermatozoa/100 µl.). The procedure was then repeated in the contralateral oviduct but injecting only BTS-diluent without spermatozoa (100 µl BTS-diluent) (Figure 1). After both oviducts were injected, the trocars were removed and minor suturing was required. The procedure involved minor surgery, which was carried out in less than 15 minutes.

### Samples collection, Embryo evaluation and RNA preparation

Uterine horn and oviduct tissue were collected from both sides of the reproductive tract in each animal by hysterectomy at day 2, 3, 4, 5 and 6 after laparoscopic inseminations. Donors were sedated by azaperone administration (2 mg/kg body weight, i.m). General anaesthesia was induced with sodium thiopental (7 mg/kg body weight, i.v) and maintained with isofluorane (3.5–5%). The reproductive tract was exposed via a mid-line incision and the corpora lutea were counted in both ovaries. Embryos (from inseminated side) and oocytes (from non-inseminated side) were collected by flushing each oviduct and uterine horn with Phosphate Buffered Saline medium (PBS, 30 ml). The presence of embryos in one side (insemination side), and the existence of unfertilized oocytes in the other side (non-inseminated side) were verified by careful examination of oviduct and uterine flushings under a stereomicroscope. Embryos were collected at different developmental stages and from different locations depending on the day of the recovery after laparoscopic insemination. At day 2, 2cells stage embryos were recovered from the oviduct; at day 3 to 4, 4cells stage embryos were recovered from the oviduct or the uterine horn or both; at day 5, morula stage embryos were recovered from the uterine horn and at day 6, blastocyst stage embryos were recovered from the uterine horn. In cases where insemination had failed, or embryos were not at the expected developmental stage for the given time point, the samples were discarded.

After evaluating embryos and oocytes in each oviduct and uterine horn and verifying that they are in the right stage of development, the reproductive tract was excised by hysterectomy operation and the uterine horn and the oviduct were opened longitudinally. Then, epithelial cells were isolated by scraping of the mucosal epithelial layer from the oviduct or the tip of the uterine horn (first 10–20 cm of the horn adjacent to utero-tubal junction) with a glass slide. Scraped cells from the uterine horn and oviductal samples were transferred immediately to Tri Reagent (Sigma, Sigma-Aldrich Co, Madrid, Spain), homogenised, snap-frozen in liquid nitrogen and stored at −80°C until further processing.

Total RNA was isolated using a standard procedure involving phenol:chloroform extraction followed by ethanol precipitation. The quantity (NanoDrop 1000 spectrophotometer) and the quality (Agilent 2100 Bioanalyser; Agilent Technologies) of the RNA samples were analysed. Only samples with satisfactory quality as indicated by the absence of degradation of the ribosomal RNA were used for microarrays and quantitative Real-Time Reverse Transcriptase-Polymerase Chain Reaction (qPCR). qPCR analyses. All samples used for the microarray experiment showed an RNA integrity number (RIN) between 7 and 9 by Bioanalyser.

### Microarrays Hybridization

Affymetrix Porcine Genome gene expression arrays (Affymetrix, Santa Clara, CA) were used in this study. Total RNA samples were prepared according to the Affymetrix Technical Manual (www.affymetrix.com). Briefly, 5 µg of total RNA was converted into cDNA using an oligo(dT) which also carries the binding site for T7 RNA polymerase. Superscript II (Affymetrix) was used to carry out this reaction. After first strand synthesis, residual RNA was degraded by addition of RNaseH and a double-stranded cDNA molecule was generated using DNA Polymerase I and DNA ligase. This double stranded molecule was used as a substrate for the T7 RNA polymerase to produce multiple copies of the cRNA using the Affymetrix IVT labelling system. The cRNA molecules produced incorporated biotin labelled ribonucleotides, which acted as a target for the subsequent detection of hybridization, using fluorescently labelled streptavidin. 13 µg of cRNA molecules were heat fragmented and injected to the Porcine GeneChips in a hybridization solution according to the Affymetrix protocol. Hybridization took place overnight in a rotating hybridization oven at 60 rpm, 45°C for 16 hours. The GeneChip arrays were washed using the Affymetrix Fluidics Station. After washing, the GeneChip arrays were scanned using Affymetrix GC3000 scanner. The resultant images were analysed using the Microarray Suite software version 5.1 (Affymetrix). At the detection level each probe set was designated as present, absent or marginal. Only present transcripts were considered expressed. Microarray experiments were carried out according to MIAME guidelines and the complete experimental data can be obtained online from the NCBI Gene Expression Omnibus (GEO) (http://www.ncbi.nlm.nih.gov/geo/) submission number GSE33262.

### Microarray Data and Bioinformatics analysis

Microarray data analysis was performed as described by Tsai et al. [33], for Affymetrix Porcine arrays. Probe intensity values were log2 transformed and normalized with Lowess normalization. Differential expression for all six arrays was determined by estimating the presence of the embryo (insemination effect) after fitting a linear mixed model using SAS and JMP/Genomics [34], as shown in [1].
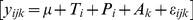
(1)For each probe set, *y* is the log2 transformed intensity of the *i* ^th^ treatment, j^th^ probe, and *k* ^th^ array. This model included a fixed effect for treatment (inseminated [presence of embryos in uterine horn] or non-inseminated [absence of embryos in uterine horn], *T*) and probe (*P*) and a random effect for array (*A*). In this equation, *μ* represents an overall mean value and ε is stochastic error. Least square means were estimated for the difference between treatments for each gene [difference of inseminated = (N—Y), where N represents non-inseminated and Y is inseminated] and p-values were adjusted with Bonferroni correction to control the error rate to >0.05.

Using Affymetrix Porcine Annotation in combination with Affymetrix Human annotation, differentially expressed transcripts from the microarray were annotated. Then, the Kyoto Encyclopaedia of Genes and Genomes (KEGG) database was used to categorize differentially expressed transcripts into different biological functions and pathways. The KEGG database contains general information on biological pathways and information on species-specific pathways [35]. A detailed description of KEGG PATHWAY database is accessible at [http://www.genome.jp/kegg/pathway.html]. When a porcine pathway was not found for a specific gene from our study, a human pathway was used instead. Data pathways from the differentially expressed transcripts were further reduced to obtain a biologically meaningful overview by organizing the transcripts into different categories and subcategories according to KEGG PATHWAY database hierarchy.

### Quantitative Real-Time Reverse Transcriptase Polymerase Chain reaction

Gene expression profiles derived from microarray analyses were confirmed using qPCR. The primers for each gene were designed, when possible, to span between adjacent exons. The primers used for qPCR are listed in Table 1. Amplified PCR products were sequenced with forward and reverse primers to verify the resulting product.

**Table 1:** Primers used for qPCR analysis.

Gene Symbol	Affymetrix Porcine Probe	Primer	Sequence	Product size (pb)
RDX	Ssc.21016.1.S1_at	Forward	5′ACACGATGAACATGATGAGA 3′	142
		Reverse	5′ACTTAATGCCTGGAGTTGCT 3′	
SLCO1A2	Ssc.16331.1.S1_at	Forward	5′GGAAGCTTTGAGATTGGAAA 3′	156
		Reverse	5′TTCATATCGGTTCATGAGGA 3′	
TICAM2	Ssc.2201.2.S1_at	Forward	5′ ACCTTCACAGCCTCCAAAAA 3′	141
		Reverse	5′ AGCACTGACTCGGTTCTCAC 3′	
β-actin	Reference gene	Forward	5′CCTCCCTGGAGAAGAGCTA 3′	152
		Reverse	5′CTTCATGATGGAGTTGAAGGT 3′	

Total RNA from the oviduct and the uterine horn samples (inseminated and non–inseminated) was treated three times with DNase I (DNA-free kit; Ambion.) to remove genomic DNA contamination from samples. First-strand cDNA synthesis was performed using High Capacity cDNA Reverse Transcription Kit (Appied Biosystems). Negative controls were prepared without inclusion of the enzyme (non-reverse transcription controls, RT controls). Reverse transcriptase PCR (RT-PCR) was performed according manufacturer instructions. To separate PCR products 10 µl of each sample was resolved on a 1.2% agarose gel and electrophoresis was performed with 1× TAE buffer and a voltage of 110 V for 40–50 min. The bands were visualized by using an ultraviolet transillumination, and digital images were obtained.

SYBR Green Jump Start (Sigma) master mix (containing 10 µl SYBR Green, 7 µl H2O, 1 µl of forward and reverse primers and 1 µl cDNA) was added to each well of PCR plate and amplification was performed under the following conditions: 40 cycles of 95° for 30 s, 55° for 1 min and 72° for 1 min. Samples without template and RT controls (without the addition of enzyme) for each primer set were included to identify contamination. Triplicate measurements for each group of samples were carried out. Quantitative PCR was performed using Mx3005P QPCR (Stratagene, Waldbronn, Germany). The quantification data were analyzed using MxPro QPCR software version 4.01. Quantitative PCR data were analyzed using the comparative CT method. Briefly, the difference in cycle times, ΔCT, was determined as the difference between the tested gene and the reference ß-actin. Then, ΔΔCT was obtained by finding the difference between groups [36]. The results were expressed as mean±SEM arbitrary gene expression values, normalized on the basis of β-actin expression. Statistical analysis was performed using analysis of variance (ANOVA); values were compared using the Bonferroni test (in SPSS, version 14.0 (SPSS Inc., Chicago, IL)). The threshold for significance was set at p<0.05.

### Mathematical modelling of the TLR4 signalling pathway using microarray data

**Figure 2:**
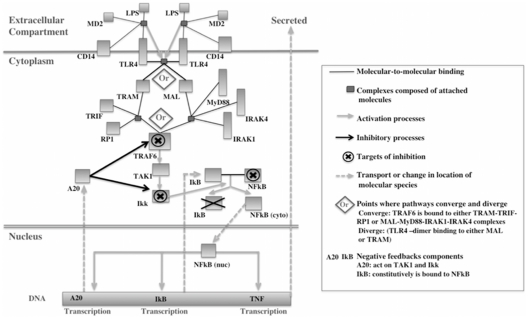
Schematic diagram of the components of TLR4 pathway model. This figure shows a schema of the components and interaction of the published TLR4 computational model from An and Faeder model [35].

The concerted effect of the transcriptional changes observed in this study has been systematically examined using mathematical modelling. In particular, a published model of TLR-4 signalling [28] was used onto which the microarray data were mapped. This is a generic model that simulates the cell response to stimulation by lipopolysaccharide (LPS) administration as measured by production of tumor necrosis factor (TNF). We fully acknowledge that the current state of development of published mathematical models falls far short of a comprehensive interpretation of global transcriptional data such as that collected in this study. The handful of mathematical models [26]–[28] that have been published are rather narrow in their focus, with only a subset of the signalling proteins, simplified kinetics and assumed parameter values. Nevertheless, we believe that this study contributes a small step towards closing the gap between theoretical modelling and experimental measurements, which is an essential aim of molecular systems biology over the coming years. Figure 2 shows a schematic diagram of the components of TLR4 pathway model from An and Faeder model [28]. In order to simulate the possible effect that the presence of the embryo might have on the immune response we first carried out a sensitivity analysis on the model. This sensitivity analysis showed how the abundance of each protein on the TLR-4 pathway influences the size of the immune response – in particular the peak concentration of TNF. Then sensitivity coefficient for each protein was calculated as the percentage change in peak height caused by a 1% increase in the concentration of that protein. Note that most proteins had a positive sensitivity - i.e. they had a positive effect on the immune response – but there were a few proteins that had negative sensitivities indicating that they had an inhibitory effect on the immune response.

Further model simulations were carried out assuming that the relative changes in the transcript levels from microarray data in the current investigation gave the same relative changes in the corresponding protein levels. The response of the uterine horn in the presence of the oocytes (non-inseminated) was considered as the base concentration (base = oocyte presence). In order to account for the high noise level in the transcript data we compared the base case (oocyte presence) to 3 scenarios of altered signalling levels in the presence of embryos in the uterine horn. These 3 scenarios represented the smallest, average and largest response that were possible given the variability of the data. This approach utilises the raw microarray data, which, for each animal, contains signals for one, or more probes that bind to the mRNA of each signalling protein in the TLR immune response pathway (see raw data in GEO submission number GSE33262).

To illustrate this approach consider the two Affymetrix probes: ‘Ssc.17204.1.S1_at’ and ‘Ssc.18913.1.S1_at’ that bind specifically to MAL - one of the signalling proteins in the TLR immune response pathway. Since three animals were used in this study there are a total of 6 measurements for the ratio of the inseminated to non-inseminated signal (i.e. 2 probes multiplied by 3 animals). These values are as follows: probe1/animal1 = 3D 0.959, probe1/animal2 = 3D 1.301, probe1/animal3 = 3D 0.848, probe2/animal1 = 3D 1.270, probe2/animal2 = 3D 0.7956, probe3/animal3 = 3D 1.511. It can be seen that there is considerable variability in this data -there are 3 ratios greater than unity and 3 less than unity with no obvious difference between probes or animals. As such, there is no statistically significant difference in expression level for this protein, but we were interested in exploring the effect of the maximum/minimum expression changes in the computational model.

For the model scenarios we used the minimum, maximum and geometric mean of these 6 values to compare the inseminated immune response with the non-inseminated response. MAL has an inhibitory effect on the immune response - i.e. the model response decreases with increasing MAL (negative sensitivity- see Table 2). We used the maximum of the 6 measurements (1.51) in the minimum response scenario; the minimum of these measurements (0.74) in the maximum response scenario; and the geometric mean of these measurements (1.08) in the average response scenario. This approach was adopted for all proteins present in the computational model which could be mapped to a gene in the microarray data.”

**Table 2:** Relative expression changes derived from the microarray data that were applied to TLR4 computational model.

Protein species	Base concentration^a^	Sensitivity	Relative expression in 3 scenarios^b^		
			*Minimum response*	*Maximum response*	*Average response*
CD14	10000	2.31E-04	3100 (0.31)	60700 (6.07)	11000 (1.10)
MD2	10000	1.87E-04	10100 (1.01)	11000 (1.10)	10600 (1.06)
TLR4	10000	1.76E-03	2600 (0.26)	32900 (3.29)	12000 (1.20)
TRAM	10000	1.19E-02	5200 (0.52)	10500 (1.05)	7800 (0.78)
MAL	10000	−1.16E-02	15100 (1.51)	7400 (0.74)	10800 (1.08)
TRIF	285	−3.40E-04	285 (1.00)	285 (1.00)	285 (1.00)
MyD88	285	9.09E-05	290.7 (1.02)	316.35 (1.11)	302.1 (1.06)
RP1	285	−3.41E-04	467.4 (1.64)	153.9 (0.54)	233.7 (0.82)
IRAK1	1100	3.08E-05	88 (0.08)	1793 (1.63)	704 (0.64)
IRAK4	285	6.23E-06	285 (1.00)	285 (1.00)	285 (1.00)
TRAF6	1100	4.74E-03	913 (0.83)	2882 (2.62)	1881 (1.71)
TAK1	10000	1.25E-01	6700 (0.67)	13400 (1.34)	9600 (0.96)
Ikk_Complex	10000	3.01E-01	6500 (0.65)	17300 (1.73)	9800 (0.98)
IkB-alpha	31.6	−1.45E-03	56.564 (1.79)	24.016 (0.76)	31.916 (1.01)
NFkB	31.6	3.24E-03	4.108 (0.13)	173.8 (5.50)	46.768 (1.48)
IRAK4_MyD88	812	1.01E-04	828.24 (1.02)	901.32 (1.11)	860.72 (1.06)
RP1_TRIF	812	−2.22E-03	1331.68 (1.64)	438.48 (0.54)	665.84 (0.82)
IkB_NFkB	9970	4.60E-01	997 (0.10)	98204.5 (9.85)	14855.3 (1.49)
IRAK1_IRAK4_MyD88	8900	5.13E-04	712 (0.08)	14507 (1.63)	5696 (0.64)
RP1_TRIF_TRAF6	8900	1.50E-02	4005 (0.45)	38270 (4.30)	12460 (1.40)

a Data expression shows the response of the uterine horn in the presence of the oocytes as the base concentration (Molecules per cell).

b Relative expression in 3 scenarios (smallest, average and largest response) of altered signalling protein levels in the presence of embryos in the uterine horn.

For each transcript of interest, the maximum and minimum expression change across all 3 animals and across all of the probes binding to the corresponding transcript were used in the analysis. Which of these expression levels we used for the maximum and minimum response scenarios depended on the sign of the sensitivity of the response of each signalling protein. If the response showed a positive sensitivity then the maximum expression change was used in the maximum response scenario but if the sensitivity was negative, then the minimum expression change was used in this scenario. Similarly, the minimum response scenario was assembled from the minimum expression changes for those exhibiting positive sensitivities and the maximum expression changes for those with negative sensitivities.

Finally, as well as estimating the expression levels of single proteins from the data (as discussed above), we also had to estimate the expression levels of those protein complexes that had non-zero initial concentrations in the An and Faeder model [28] (e.g. IRAK4_MyD88 which represents the hetero-dimer formed by IRAK4 bound to MyD88). Clearly, the microarray data only provided expression levels for individual proteins rather than complexes. This problem was overcome by multiplying the relevant expression levels of the constituent protein species. This was reasonable since it assumed an equilibrium between the dimer and the two constituent proteins that comprise it.

### Experimental design

We hypothesized that the presence of the embryo at different stages of development elicits a local response in the oviduct and/or uterine horn that is only present in the oviduct or the horn that contains the embryo and is absent in the horn that contains oocyte. To test our hypothesis a novel experimental model design was used. Each sow was subjected to laparoscopic insemination. While one oviduct was inseminated with spermatozoa (inseminated side, presence of embryos) the contralateral oviduct was injected only with diluent (BTS) (non-inseminated side, presence of oocytes). Figure 1 provides a schematic overview of the experimental model employed.

Microarray and qPCR were used to analyze gene expression of the oviduct and the uterine horn containing embryos at different embryo developmental stage and oocytes. For this purpose, different experiments were designed.

#### Does the blastocyst induce a local response in the uterine horn?

We compared the gene expression profile of the uterine horn containing embryos to that of oocytes using microarray technology. We hypothesised that if blastocysts induce a local response in the uterine horn, the gene expression profiles of the uterine horn containing embryos would be different from that containing oocytes in each individual sow. Uterine horn samples containing embryos at the blastocyst stage and their contralateral uterine horn containing oocytes from individual sows were used. Three biological replicates were performed (n = 3 sows) and a total of 6 arrays were used for microarrays study (3 arrays for uterine horn samples containing embryos (inseminated-side) and 3 arrays for samples containing oocytes (non-inseminated side)).

After performing microarray experiments and bioinformatic data analysis, three transcripts were selected to validate the results: RDX (radixin; Affymetrix probe Ssc.21016.1.S1_at; G = Ssc.21016), SLCO1A2 (solute carrier organic anion transporter family, member 1A2; Affymetrix probe Ssc.16331.1.S1_at, AF403248.1) and TICAM2 (TIR domain- containing adapter molecule 2; Affymetrix probe Ssc.2201.2.S1_at; BX670675). Recent studies have shown the expression of these genes in the uterus and the placenta during implantation and late pregnancy. These evidences lead us to select these genes in our study [37]–[42]. For this experiment, 3 biological replicates (n = 3 sows containing embryos at blastocyst stage) and 3 technical replicates were performed.

#### Spatio-temporal analysis of mRNA levels of a selected transcript: Does the embryo developmental stage affect the local response of the oviduct and uterine horn?

Based on the microarrays results one specific transcript, TICAM2, was selected for the spatio-temporal study. Up to now little is known about the role of TICAM2 in the very early stages of pregnancy. The mRNA levels of TICAM2 were analyzed using qPCR from the oviducts and the uterine horns containing embryos at different stages of development (2cells, 4cells, morula and blastocyst). For this experiment, 2–3 biological replicates (3 sows per 2 cells stage; 2 sows for 4 cells in the oviduct; 3 sows for 4 cells in the uterine horn; 2 sows for morula stage and 3 sows for blastocyst stage) and 3 technical replicates were performed.

#### Does the embryo induce a local regulation of the immune system in the uterine horn?

A mathematical model based on a published model of TLR-4 signalling was mapped onto the microarray data to evaluate the potential effect of the presence of the blastocyst on the regulation of a key pathway for innate immunity in the uterine horn.

## Results

### The blastocyst induced a local response in the uterine horn compared to oocyte

**Figure 3:**
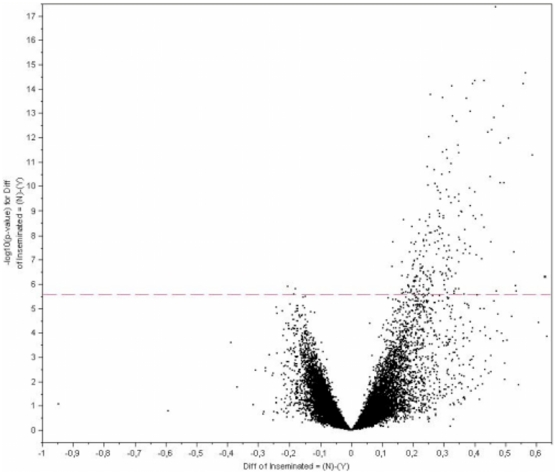
Mapping of differentially expressed genes onto Volcano plot. A Volcano plot depicting significant changes in gene expression between uterine horn in the presence of the blastocyst (Inseminated side) and uterine horn in the presence of oocytes (Non inseminated side). Each of the 23,124-oligonucleotide probes is plotted and probes showing significant differences in gene expression (210 probes, p<0.05) are above the red broken line.

Microarray analysis revealed that around 1% of transcripts (210 out of 24123 probes from Affymetrix Porcine Chip) were consistently altered (P-value<0.05) in the uterine horn in the presence of embryos at the blastocyst stage compared to the presence of oocytes. Most of the transcripts were down-regulated (208) and only 2 transcripts were up-regulated when the blastocyst was present in the uterine horn. Figure 3 provides a visualization of the significant changes in transcript expression values between the two groups of samples. A complete list of transcripts (genes and ESTs) altered in the uterine horn samples in the presence of blastocysts compared to oocytes is presented in Dataset S1.

**Figure 4:**
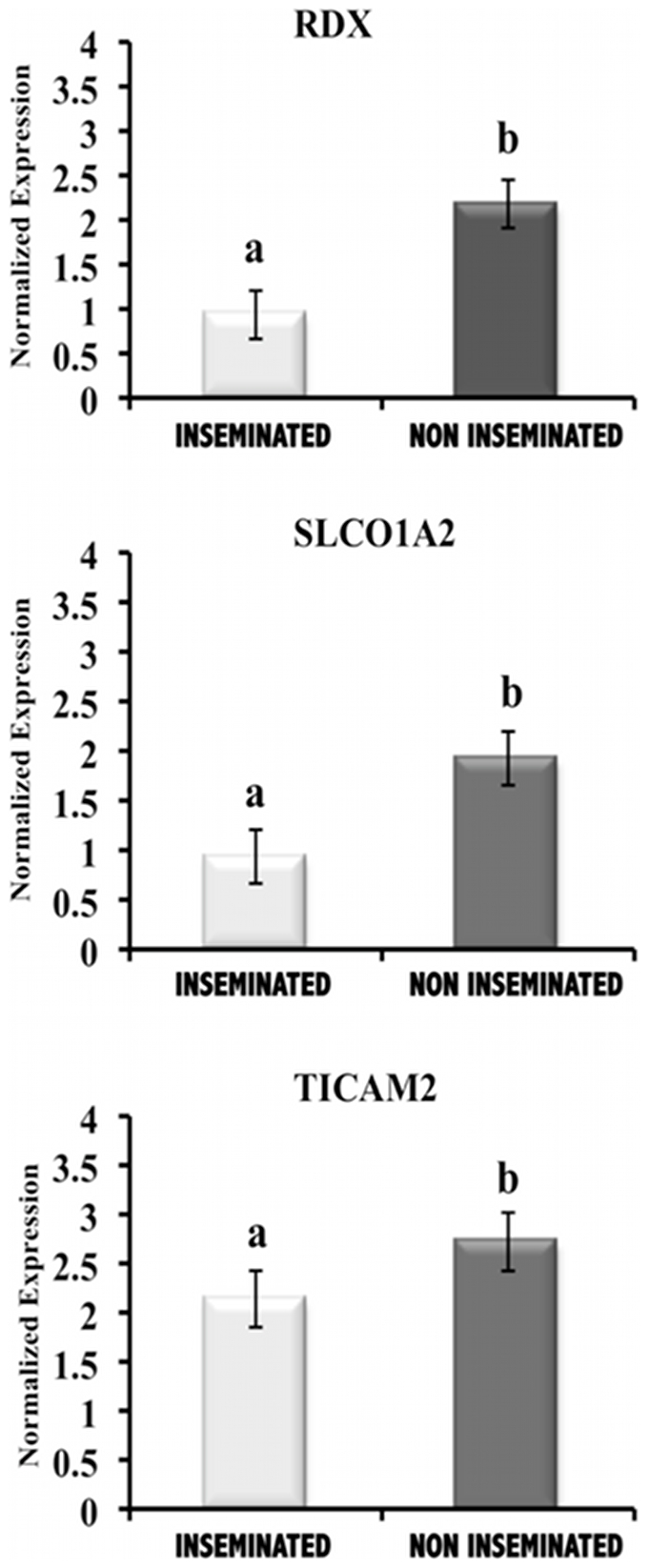
Validation of the microarray results by qPCR analysis. RDX (radixin), SLCO1A2 (solute carrier organic anion transporter family member 1A2) and TICAM2 (TIR domain- containing adapter molecule 2) expression values (normalized based on ß–actin expression values) in uterine horn samples in the presence of embryos at the blastocyst stage (Inseminated) compared to the presence of oocytes (Non Inseminated). The expression of all transcripts in the uterine horn in the presence of blastocyst was significantly different from that of oocyte in the uterine horn (P<0.05).

Significant differences observed in the gene expression of RDX, SLCO1A2 and TICAM2 using microarray data analysis were validated by qPCR. The presence of blastocysts in the uterine horn resulted in significant decreases (P<0.05) in the gene expression values of RDX, SLCO1A2 and TICAM2 transcripts when compared to the existence of oocytes in the uterine horn (Figure 4). The qPCR analysis clearly confirmed the microarray findings.

### Altered transcripts function and biological pathways identification

From 210 transcripts undergoing changes in the presence of blastocyst compared to oocytes, 84 transcripts (40%) were genes and ESTs with known biological function in the KEGG database. These 84 transcripts were found to be active in a total of 99 pathways, since one transcript may be involved in more that than one pathway. For example we found that the focal adhesion pathway displayed 7 transcripts that were differentially expressed in our study. However, most of the transcripts were involved in only a single pathway. More information regarding the number of transcripts involved in each pathway can be found in Dataset S2.

**Figure 5:**
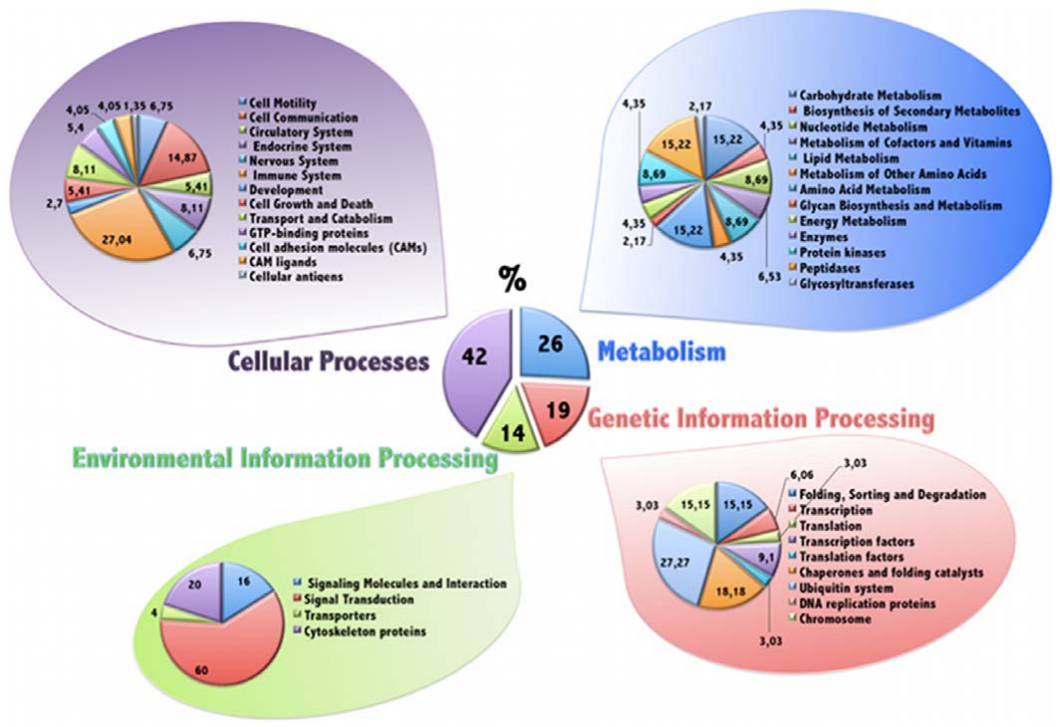
Transcripts differentially expressed in uterine horn in presence of embryos organized into functional categories. The proportions of the differentially expressed transcripts were organized into 4 major categories (first level) and different subcategories (39) (second level) on the basis KEGG PATHWAY database hierarchy The central pie chart represents the first level of organization and the lateral pies display the second level of organization: a total of 39 subcategories containing the 84 different pathways associated with the differentially expressed transcripts.

Data pathways from the differentially expressed transcripts were further reduced to obtain a biologically meaningful overview by organizing the transcripts into different categories and subcategories according to KEGG PATHWAY database hierarchy. The proportions of the differentially expressed transcripts were organized into the 4 major categories (first level) and different subcategories (39) (second level) by KEGG are shown in Figure 5. The largest subcategories were involved in signal transduction, cell communication and immune system. The pathways in which altered transcripts were involved with are presented in Table 3. Other interesting subcategories with high numbers of transcripts involved were: Carbohydrate Metabolism, Amino Acid Metabolism, Peptidases, Endocrine System, Ubiquitin system and Transport and Catabolism. The results of all data pathways classification are available in Dataset S2.

**Table 3:** Differentially expressed transcripts and pathways involved in Signal transduction, Cell communication and immune system.

KEGG* Category	KEGG Subcategory	KEGG Pathways	Transcripts
*Environmental Information Processing*	Signal Transduction	Calcium signaling pathway	VDAC1, PPP3CB
		VEGF signaling pathway	PPP3CB
		Wnt signaling pathway	MAPK9, WIF1, PPP3CB, PPP2R5E,
			ROCK1
		TGF-beta signaling pathway	ROCK1
		MAPK signaling pathway	PPP3CB, CASP3, RAP1A, MAPK9,
		ErbB signaling pathway	MAPK9,
		Phosphatidylinositol signaling system	IMPA2
*Cellular Processes*	Cell Communication	Gap junction	GJA1
		Tight junction	CLDN10, YES1
		Adherens junction	YES1
		Focal adhesion	ROCK1, CAV2, RAPIA, MAPK9,
			ARHGAP5, PPP1R12A, PPP1CC
	Immune System	Complement and coagulation cascades	CD55
		Hematopoietic cell lineage	CD55
		Toll-like receptor signaling pathway	TICAM2, MAPK9
		NOD- like receptor signaling pathway	ERBB2IP, MAPK9
		RIG-I-like receptor signaling pathway	MAPK9
		B cell receptor signaling pathway	PPP3CB
		T cell receptor signaling pathway	MAPK9, PPP3CB,
		Fc epsilon RI signaling pathway	MAPK9
		Fc gamma R-mediated phagocytosis	ARPC1A
		Natural killer cell mediated cytotoxicity	CASP3, PPP3CB
		Leukocyte transendothelial migration	ARHGAP5, CLDN10, RAP1A, ROCK1
		Chemokine signaling pathway	RAP1, ROCK1

* Differentially expressed transcripts organized into the more representative functions and the sub-pathways in which are involved according to KEGG (Kyoto Encyclopedia of Genes and Genomes) database.

### Spatio-temporal pattern of TICAM2 expression: The response of the oviduct and the uterine horn towards the embryo is dependent on the embryo developmental stage

**Figure 6:**
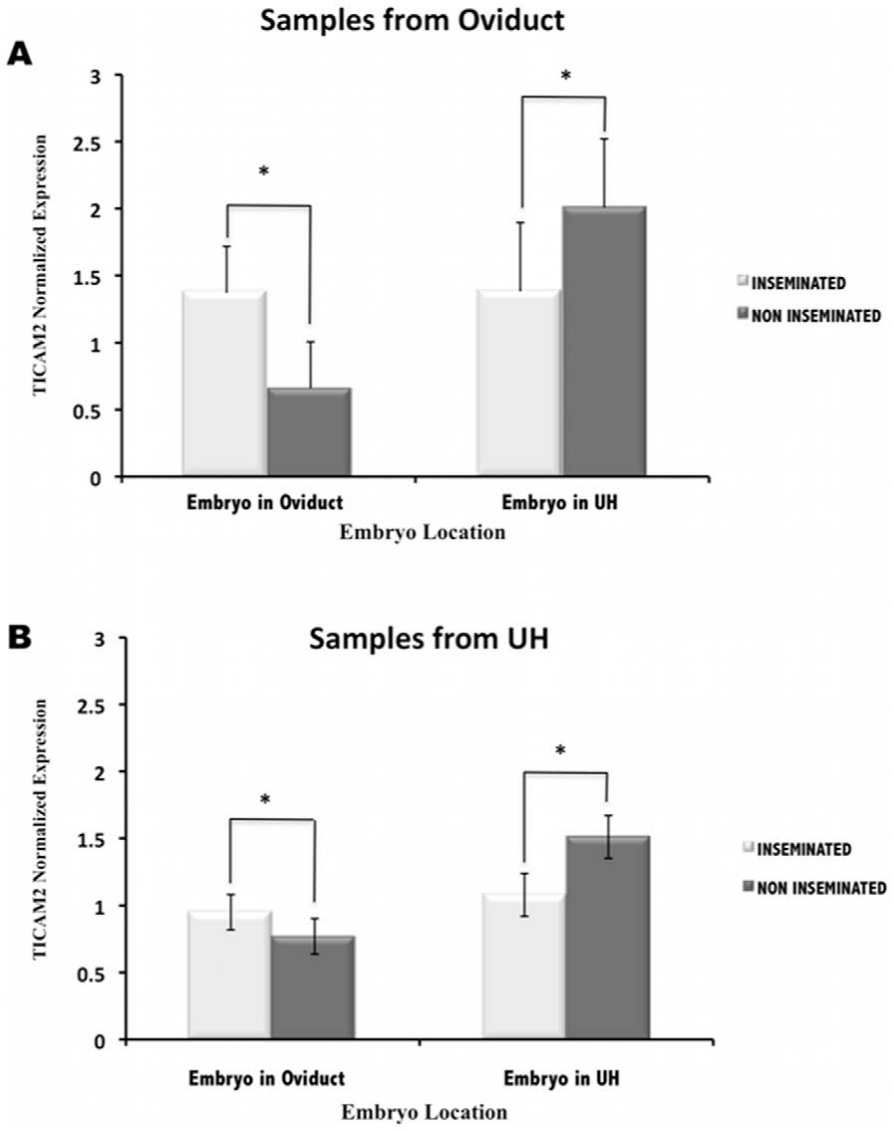
Spatial–course of TICAM2 expression in oviduct and uterine horn (UH) samples. TICAM2 expression values (normalized based on ß–actin expression values) in oviduct (**A**) and UH samples (**B**) in the presence of embryos at different stages of development (Inseminated) and in the presence of oocytes (Non inseminated). Embryo was located in oviduct at 2 cells and 4 cells embryo stage; Embryo was located in uterine horn at 4 cells, morula and blastocyst). *(P<0.05).

TICAM2 was selected for a further detailed spatio-temporal expression analysis. Quantitative PCR analyses showed that embryo location influenced the transcriptome expression in the oviduct and the uterine horn (Figure 6). The analysis of TICAM2 expression from both the oviduct (Figure 6A) and the uterine horn (Figure 6B) samples revealed that the presence of the embryo in the oviduct up-regulated this gene when compared to oocytes. In contrast, when the embryo migrated from the oviduct to the uterine horn, TICAM2 expression was down-regulated in both the oviduct (Figure 6A) and the uterine horn samples (Figure 6B).

**Figure 7:**
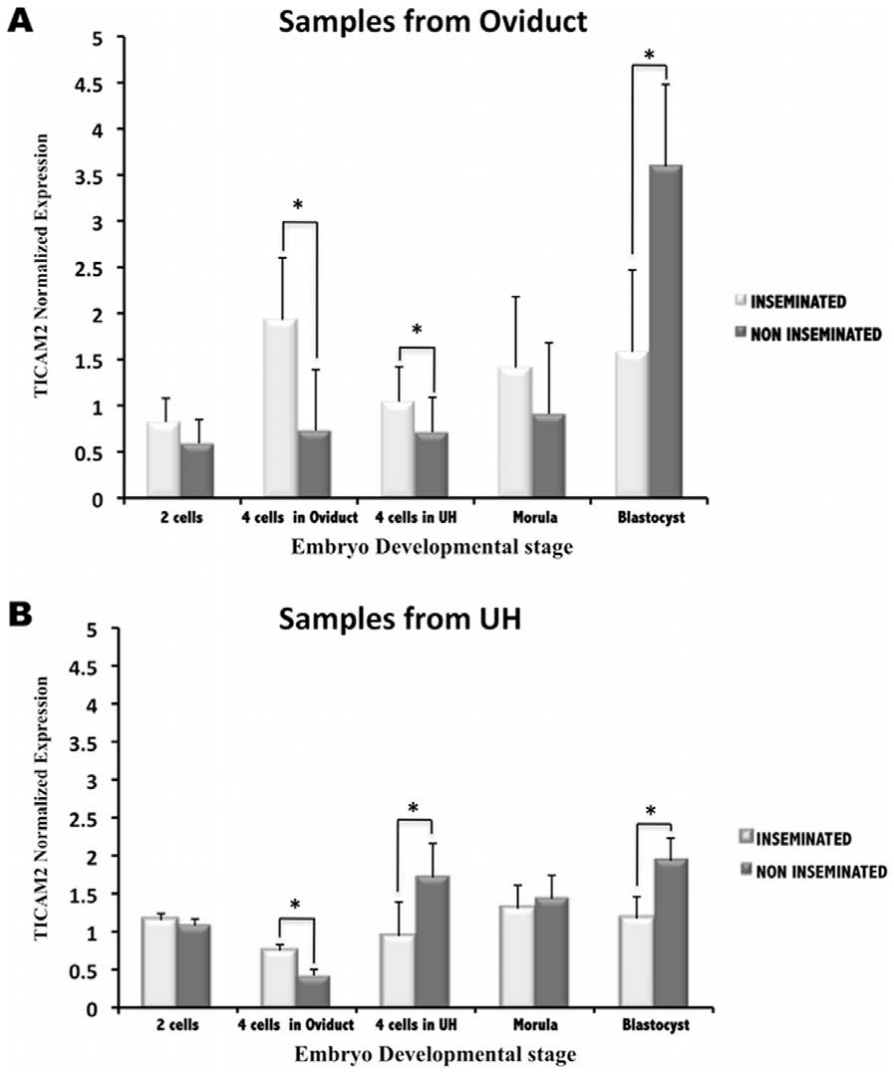
Time–course of TICAM2 expression in oviduct and uterine horn (UH) samples. TICAM2 expression values (normalized based on ß–actin expression values) in oviduct (**A**) and UH samples (**B**) in the presence of embryos at different stages of development (Inseminated) and in the presence of oocytes (Non inseminated). *(P<0.05).

Additionally, our results revealed that the TICAM2 expression pattern was dependent on the embryo developmental stage (Figure 7). TICAM2 expression from both the oviduct and uterine horn samples was significantly different (P<0.05) between inseminated and non-inseminated samples when embryos were at the 4cells stage and the blastocyst stage. Oviductal samples showed a down-regulation of TICAM2 expression in the presence of the embryo at the blastocyst stage when compared to previous embryo stages of development where TICAM2 was up-regulated (Figure 7A). However, when TICAM2 expression was analysed in the uterine samples, TICAM2 expression shifted from up-regulated to down-regulated when the embryo migrated from the oviduct to the uterine horn at the 4cells stage. This down-regulation remained until the blastocyst stage (Figure 7B).

### *In silico* modelling

The *in silico* model consisted of 76 molecular species, which are mainly individual proteins or protein complexes and a total of 203reactions that produced or consumed these species as defined by the reaction stoichiometry. The dynamic mass balance on each of these species (rate of accumulation = total rate of production – total rate of consumption) were, in mathematical terms, written as a set of ordinary differential equations (ODEs). These equations were integrated to show how the molecular species varied over time for different levels of stimulus or different expression levels of the intermediate signalling proteins. This was a standard approach but, in common with other signalling models, the lack of measured parameter values (rate constants and protein abundances) means that such a model generally made only qualitative rather than truly quantitative predictions [27], [28].

The sensitivities of the immune response to the TLR pathway signalling proteins are shown in table 2. Most proteins had a positive sensitivity - i.e. they had a positive effect on the immune response. A few proteins (e.g. for MAL) had negative sensitivities indicative of their inhibitory effect on the immune response. This implies that increasing the concentration of these proteins actually reduces the immune response.

IkB_NFkB heterodimer, the IKK complex and the single protein TAK1 had the highest sensitivity in the *in silico* model (table 2). Table 2 also shows the relative expression changes derived from the microarray data that were applied to the base concentrations (oocyte presence) in each of the 3 scenarios of altered signalling protein levels in the presence of embryos in the uterine horn (smallest, average and largest response).

**Figure 8:**
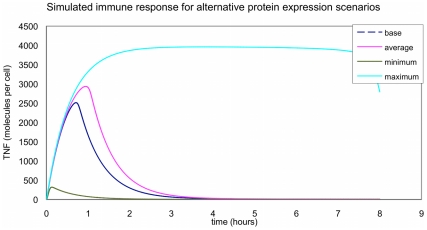
TNF profiles for the different scenarios. ****The figure shows the different response of the model in terms of produced TNF in the presence of oocytes (base) and embryos (maximum, average and minimum response).

The predicted TNF profiles for the different scenarios are shown in figure 8. The average response in the presence of the embryo had a peak approximately 15% higher than the base (oocyte presence) whereas the smallest response in the presence of the embryo had a peak that was only 13% of the base peak (oocyte presence). The largest response in the presence of the embryo had a peak that was 57% bigger than the base peak and much longer response duration (the peak interval was over 5 times that of the base case (oocyte presence).

## Discussion

The results of the present study support our hypothesis that the embryo elicits a local response in the uterine horn, modulating the maternal response in different biological functions and pathways. In this model uterine horns containing embryos and oocytes were exposed to the same hormonal milieu. Hence, alterations observed in the endometrial cell expression profiles can only be the result of the local embryo interaction with the endometrium.

Different model approaches have previously been used to study the molecular changes that occur in the endometrium in pregnant and not-pregnant samples. A monozygotic twin model has been used to address the differences in endometrial gene expression between pregnant and cycling animals on day 18 in cattle [16], [17]. This model offers the genetic uniformity of monozygotic twin pairs, reducing the effects of genetic variability, a factor that potentially affects the results of gene expression analyses [43]. However, in this model the endometrium from the pregnant animals is compared to cycling animals. Therefore, differences observed in the gene expression of the endometrium could be due to maternal hormones associated with pregnancy, the presence of embryos or both.

In an attempt to study the local changes produced in the oviductal transcriptome by the presence of the embryo, Lee and co-workers [10], used a similar model to the one we used in our investigation. In this model, 1-cell embryos and oocytes were collected from different mice and were transferred to opposite oviducts in the same pseudo-pregnant female mice. Although this model allowed exposing embryos and oocytes to the same milieu after the transfer, both embryos and oocytes were totally allogenic, whether to the pseudo-pregnant mice or to each other. This is different from what happens in a normal pregnancy, where the embryo is a semi-allogenic entity. In the current investigation we compared the endometrium transcriptome profile in response to embryos and oocytes from the same sow without any potential interference that may be brought to the experimental design through the use of oocyte and embryos from another individual. In addition, in the present study the uterine response to embryos and oocytes took place under the same hormonal conditions.

Our findings suggested that the presence of the embryo leads to critical changes in the uterine horn, which are required for the development of a receptive maternal environment. Microarray analysis showed that most of the transcripts differentially expressed in this study were down-regulated in the uterine horn in response to blastocysts when compared to that of oocytes. RDX and SLCO1A2 gene expression was further confirmed using qPCR. We selected RDX and SLCO1A2 to validate our results because they play important roles during pregnancy [37]–[40], [44]. However, no studies have examined the expression of these genes in the oviduct and uterus during the very early stages of pregnancy. RDX is a member of Ezrin/Radixin/Moesin (ERM) family, which are involved in the construction of the cellular architecture necessary for blastocyst activation and uterine receptivity, leading to successful implantation [37]. An *in vitro* study reported that moesin and ezrin expression must be decreased for increased endometrial receptivity [38]. In the present study, RDX expression was down-regulated by the presence of the embryo in the uterine horn, indicating that it might be involved in dynamic changes in the endometrium under the presence of embryo. SLCO1A2 is a member of the solute carrier (SLC) group of membrane transport proteins. The down-regulated expression of SLCO1A2 in the presence of blastocysts when compared to oocytes might indicate different needs for embryos and oocytes in the uterine horn. Different reports indicating different levels of expression of SLCO1A2 and other members of the SLC family throughout gestation in the placenta and the embryo may suggest that the oocyte and embryo at each developmental stage express a unique set of transporters depending on the nutrients required [39], [40], [44]. Further studies are needed to clarify the role of RDX and SLCO1A2 in the embryo-maternal interface.

When genes that were differentially expressed in our study in the presence of embryos in the uterine horn were grouped into general categories and pathways the largest changes in gene expression were related to signal transduction, cell communication and immune system, which is in agreement with previous reports [11], [16], [17]. Moreover, immune related genes were the most representative of the altered genes in the present study. It is logical to assume that the maternal immune system is one of the key regulators of pregnancy. Under normal circumstances, when the maternal tract is exposed to pathogens or a non-self entity, the immune system responds in an aggressive manner. However, during pregnancy the embryo, which is a semi non-self entity is accepted in the maternal tract, nourished and carried to term. Hence, disturbances of the immune system during early pregnancy leading to disruption of this tolerance may be responsible for early pregnancy failure [18].

Walker and co-workers [18] suggested that the up-regulation of immune related genes might protect the embryos against infection during pregnancy and induce immune tolerance to the embryo. In contrast, we found that all of the immune related genes identified in our study were down-regulated in the presence of embryos when compared to oocytes. Our results were in agreement with Reese and co-workers, who reported that a set of immune related genes were down-regulated in the presence of embryos at the site of implantation [45]. Reese and co-workers pointed out that this could be one of the mechanisms whereby the embryo escapes the maternal immune response. In view of our findings we can suggest that the embryo plays a role as a modulator of the immune system in the maternal tract. But how the exact modulation of the innate immune system is coordinated needs to be elucidated.

To test our hypothesis that the embryo may act as a modulator of immunity, we have used a mathematical modelling approach. The use of computational methods in biomedical research to understand complex biological signalling systems and capturing essential aspects of intracellular dynamics has increased in the recent years [46], [47]. Pregnancy is a complex immunological process, where numerous cell types and mediators act in concert in order to protect the embryo and support embryo development while maintaining maternal defence against pathogens. TLR are considered the first mechanism of defence of innate immunity against infection and also act as regulators of cytokine network involved in early stages of pregnancy [41]. Given that there is no model available for the maternal embryo interactions to date, we focused on TLR4 pathway, one of the most intensively studied inflammatory signalling pathways [48], [49]. TLRs are widely expressed at the maternal-fetal interface and their expression has been reported to vary during pregnancy [42]. Moreover, evidence from our lab have shown that stimulation of TLRs in the female reproductive tract at early stages of pregnancy may lead to implantation failure and fetal loss [50]. Here, we used a published model of TLR-4 signalling [28] to examine particular concerted changes of the transcriptional profile observed in response to the embryo when compared to oocytes. The model simulated the dynamics of TLR-4 signalling in response to LPS stimulation and this response was measured by the production of tumor necrosis factor (TNF). The model showed that a regulation of the maternal innate immune system is possible by a combination of relatively modest changes in expression of signalling proteins due to the presence of the embryo in the uterine horn compared to oocyte. The results observed in this model supported our hypothesis that the embryo plays a role as a modulator of the immune system in the maternal tract. However, further systematic research is needed combining computational modelling and global or targeted transciptomics and proteomics to fully understand the interactions between the maternal tract and the embryo.

Our microarray data together with the computational model results points to the existence of an embryo recognition system in the uterine horn that alerts the mother to the presence of the embryo much earlier than was originally reported. In porcine, it has been reported that the embryo maternal recognition signal becomes evident around day 12 of pregnancy, when the conceptus releases a surge of estrogen [51], [52]. However, our study took place when the embryo was at the blastocyst stage, at around day 6 of pregnancy, suggesting that a local response existed a long time before the estrogen from the conceptus starts signalling. This local response of the uterus towards the presence of the embryo at the blastocyst stage would result in the alteration of expression of different genes that are identified here.

In this regard, how can the maternal tract recognize the embryo(s) and alter its environment in response to them? This question becomes more complex taking into consideration the fact that that the embryo itself is a non-self entity and as such, should initiate an immune response to reject the embryo from the maternal tract. Recent findings indicate that endometrial differentiation before embryo attachment is carried out under dual control by the endocrine and immune system [53]. However, the mechanism by which the mother can recognize the embryo at the very earliest stages of pregnancy remains largely unknown. Fujiwara and co-workers proposed that the immune system could recognize the presence of the developing embryo before hatching [53]. Our results support this idea by the down-regulation of a set of immune-related genes expressed in the presence of embryo when compared to oocytes. One of the most obvious differences between oocytes and embryos are the changes in the zona pellucida (ZP) in the developing embryo. The ZP, which is a glycoprotein coat surrounding both the oocyte and the embryo, is subjected to degradation during embryo development, from fertilized eggs to hatched embryos [54], [55], remaining intact only in unfertilized eggs. In addition, it has been reported that there are changes in the surface structure of the ZP between mature oocytes and preimplantation embryos [56]. Changes in the ZP surface from a porous structure to a compact surface during *in vivo* embryo development have also been reported [57]. In contrast to an oocyte with an intact ZP, degenerated products from ZP glycoproteins might be released from embryos in the oviduct or the uterine horn; these degenerated products from the ZP would transfer information about the developing embryo to the maternal immune system. It is possible that soluble factors from the embryo were able to transverse the ZP inducing functional changes in the maternal tract [53]. Therefore, the ZP seems to play a key role for the modulation of signals exchanged between the maternal tract and embryos or oocytes [58].

The changes in the ZP during the earliest stages of embryo development gave rise to new questions. Does the response of the uterus differ when the embryo is at blastocyst stage compared to earlier stages of development? Moreover, could the oviduct recognize the presence of the embryo? To answer these questions we carried out a spatio-temporal study of a specific gene, TICAM2, in the oviduct and the uterine horn. TICAM2 was selected for this study because this gene was one of the highly altered immune related genes observed in our microarray experiment. Moreover, TICAM2 is involved in TLRs signalling pathway. It is known that different TLRs and their adaptors molecules are involved in many pregnancy disorders. However little is known about the involvement of TICAM2 in the very early stages of pregnancy. The present study showed that the TICAM2 expression pattern was dependent on the embryo development stage and the embryo location. When the embryo was still in the oviduct at 2cells and 4cells stage, the TICAM2 expression in uterine horn (UH) samples was up-regulated. The migration of the embryo at the 4cells stage to the uterine horn showed a down-regulation of this gene in UH samples that remained until the blastocyts stage. In pigs, the onset of embryonic gene transcription takes place when the embryo is at the 4cells stage [59] and also at this stage the embryo moves from oviduct to the uterus. This embryonic stage is undoubtedly a delicate time point in the embryo development. The changes experienced by the embryo during this time may affect the maternal response to the embryo. Additionally, the changes observed in the uterine horn while the embryo was still in the oviduct imply that there is a local effect of the embryo on the oviduct that extended to the uterine horn. These changes may help to prepare the uterus for the acceptance of the embryo. Wakuda and colleagues [60] reported that functional changes in the endometrium could be induced in pregnant mice even when embryo migration to the uterus was inhibited by ligation of the utero-tubal transition sites [60]. In addition, Fujiwara and collegues [53] suggested that mothers might recognize embryos even when the embryo is still in the oviduct. Therefore, it seems that embryo-maternal communication may occur in the oviduct at very early stages of embryo development.

In conclusion, this study provides a holistic view of the local changes in the uterine horn in response to the embryo at the blastocyst stage. This response suggests that embryo maternal communication exists at the very earliest stages of pregnancy; a long time before that well-known embryonic signal of maternal recognition (interferon tau or estrogen) starts signalling. In this embryo maternal communication, the embryo might play a role as a modulator of the immune system in the maternal tract, inducing the down-regulation of immune related genes to allow the refractory uterus to tolerate the embryo and support its development. This work firmly demonstrates the local effect of the embryo in the uterus and also in the oviduct and provides new insights into molecular interactions between the mother and the embryo at the very early stages.

## Supporting Information

Dataset S1 **List of candidate transcripts differentially expressed in the uterine horn in the presence of the embryos at the blastocyst stage compared to the presence of oocytes.**
(XLS)

Dataset S2 **Biological function and pathways of differentially expressed transcripts organised according to KEGG database.** **A**) Biological function of differentially expressed transcripts classified on the basis KEGG database hierarchy. The column A represents the first level of organization (central pie in Figure 5). The column B displays the second level of organization (lateral pies in Figure 5). The column C represents the transcripts differentially expressed in our study. **B**) Representation of pathways information of differentially expressed transcripts on the basis of KEGG database.
(XLS)
